# Short- and Long-Lived Autoantibody-Secreting Cells in Autoimmune Neurological Disorders

**DOI:** 10.3389/fimmu.2021.686466

**Published:** 2021-06-17

**Authors:** C. Zografou, A. G. Vakrakou, P. Stathopoulos

**Affiliations:** ^1^ Institute of Neuropathology, University of Zurich, Zurich, Switzerland; ^2^ First Department of Neurology, National and Kapodistrian University of Athens Medical School, Athens, Greece

**Keywords:** short-lived, long-lived plasma cells, IgG4, autoantibody-mediated disorders, rituximab, neurological autoimmunity

## Abstract

As B cells differentiate into antibody-secreting cells (ASCs), short-lived plasmablasts (SLPBs) are produced by a primary extrafollicular response, followed by the generation of memory B cells and long-lived plasma cells (LLPCs) in germinal centers (GCs). Generation of IgG4 antibodies is T helper type 2 (Th2) and IL-4, -13, and -10-driven and can occur parallel to IgE, in response to chronic stimulation by allergens and helminths. Although IgG4 antibodies are non-crosslinking and have limited ability to mobilize complement and cellular cytotoxicity, when self-tolerance is lost, they can disrupt ligand-receptor binding and cause a wide range of autoimmune disorders including neurological autoimmunity. In myasthenia gravis with predominantly IgG4 autoantibodies against muscle-specific kinase (MuSK), it has been observed that one-time CD20^+^ B cell depletion with rituximab commonly leads to long-term remission and a marked reduction in autoantibody titer, pointing to a short-lived nature of autoantibody-secreting cells. This is also observed in other predominantly IgG4 autoantibody-mediated neurological disorders, such as chronic inflammatory demyelinating polyneuropathy and autoimmune encephalitis with autoantibodies against the Ranvier paranode and juxtaparanode, respectively, and extends beyond neurological autoimmunity as well. Although IgG1 autoantibody-mediated neurological disorders can also respond well to rituximab induction therapy in combination with an autoantibody titer drop, remission tends to be less long-lasting and cases where titers are refractory tend to occur more often than in IgG4 autoimmunity. Moreover, presence of GC-like structures in the thymus of myasthenic patients with predominantly IgG1 autoantibodies against the acetylcholine receptor and in ovarian teratomas of autoimmune encephalitis patients with predominantly IgG1 autoantibodies against the N‐methyl‐d‐aspartate receptor (NMDAR) confers increased the ability to generate LLPCs. Here, we review available information on the short-and long-lived nature of ASCs in IgG1 and IgG4 autoantibody-mediated neurological disorders and highlight common mechanisms as well as differences, all of which can inform therapeutic strategies and personalized medical approaches.

## Introduction

B cells are the major components of the humoral adaptive immune system. Prior to antigenic stimulation, B cells develop in the bone marrow, where V (variable), D (diversity), and J (joining) gene recombination occurs, leading to the formation of the immunoglobulin antigen binding domains and the naïve B cell receptor repertoire. During this process of development and diversity generation, autoreactive clones are physiologically cleared by mechanisms imposed by two tolerance checkpoints: one central and one peripheral (1). Upon antigenic challenge, B cells of secondary lymphoid tissue are exposed to the antigen and form antibody-secreting B cells (ASC) by two complementary pathways: the first extrafollicular, and the second involving a germinal center (GC) reaction (follicular pathway) (2–4). A canonical response to a foreign antigen involves a switch from the first pathway to the second within approximately a week. The products of B cell development and differentiation—ASCs—can be divided into short-lived plasmablasts (SLPBs) and long-lived plasma cells (LLPCs). SLPBs express unswitched or isotype-switched immunoglobulin (Ig), and their formation can indicate a rapid antigen clearance response (5). In contrast, precursors of LLPCs are typically, but not always, isotype switched and upon exit from GCs either become peripheral memory B cells or enter a survival niche—such as the bone marrow—and become LLPCs.

Both SLPBs and LLPCs may contribute to the pathogenesis of neurological autoimmune diseases. Moreover, pathogenic autoantibodies produced by autoreactive ASCs and directed against neurologic antigens can either be predominantly of the IgG1 or the IgG4 subclass, or in rarer cases can be of both subclasses. Interestingly, the predominant subclass seems to be connected to whether autoantibody-secreting cells are short- or long-lived. A specific example relates to myasthenia gravis (MG) associated with predominantly IgG4 autoantibodies against muscle-specific kinase (MuSK), where autoreactive ASCs appear to be short-lived (6). This short-lived nature is supported by the observation that MuSK autoantibody titers decrease rapidly after CD20^+^ B cell depletion with rituximab (7–9). As most of the ASCs are CD20^-^ and are not directly targeted by rituximab, titer reduction can be explained by depletion of the CD20^+^ ASC-progenitor cells in combination with the short-lived nature of MuSK ASCs. In MG, however, with predominantly IgG1 autoantibodies against the nicotinic acetylcholine receptor (AChR), titer decline post rituximab shows that B cell depletion varies from minimal (9, 10) to less pronounced in comparison to MuSK MG (11–13). Hence the AChR ASCs are presumed to be more long-lived (14). Of note, clinical responses to rituximab resemble —to some extent—autoantibody titers and comprise dramatic improvement in most cases of MuSK MG (8, 9, 15, 16) but are less pronounced (yet favorable in many cases) in AChR MG (NCT02110706) (17–24).

In this review, we aim to examine whether the MG paradigm extends to other autoimmune neurologic disorders with pathogenic autoantibodies of the IgG1 and IgG4 subclass. Further, we discuss how antigen-experienced B cells differentiate into predominantly IgG1- or IgG4- secreting ASCs and how IgG1 and IgG4 B cell responses generate short- and long-lived autoantibody-producing cells differently. While focusing on human data, we give an overview of how the different subclasses and ASCs contribute to different autoimmune neurological diseases, and in parallel, highlight advances in B cell biology that relate to the development of pathogenic autoantibodies.

## Short- and Long-Term Humoral Immunity in Infection, Allergy, and Autoimmunity

With the exception of IgM autoantibodies against myelin-associated glycoprotein (MAG) (25), all pathogenic autoantibodies against neurologic cell surface protein antigens are class-switched immunoglobulins (IgG). In turn, presence of IgG is typically, but not always, associated with GC responses and the formation of long-lasting immunological memory. With the current wealth of information about the development of humoral responses, we can better appreciate the cellular and molecular mechanisms that lead to the generation of such responses to pathogens, as well as to allergens and autoantigens. In the following sections, we will briefly summarize current concepts of B cell responses, general rules and their exceptions, and further explain how these might be relevant to the formation of neurologic autoantibodies of the IgG1 and IgG4 subclass.

### Extrafollicular B Cell Responses

**Figure 1:**
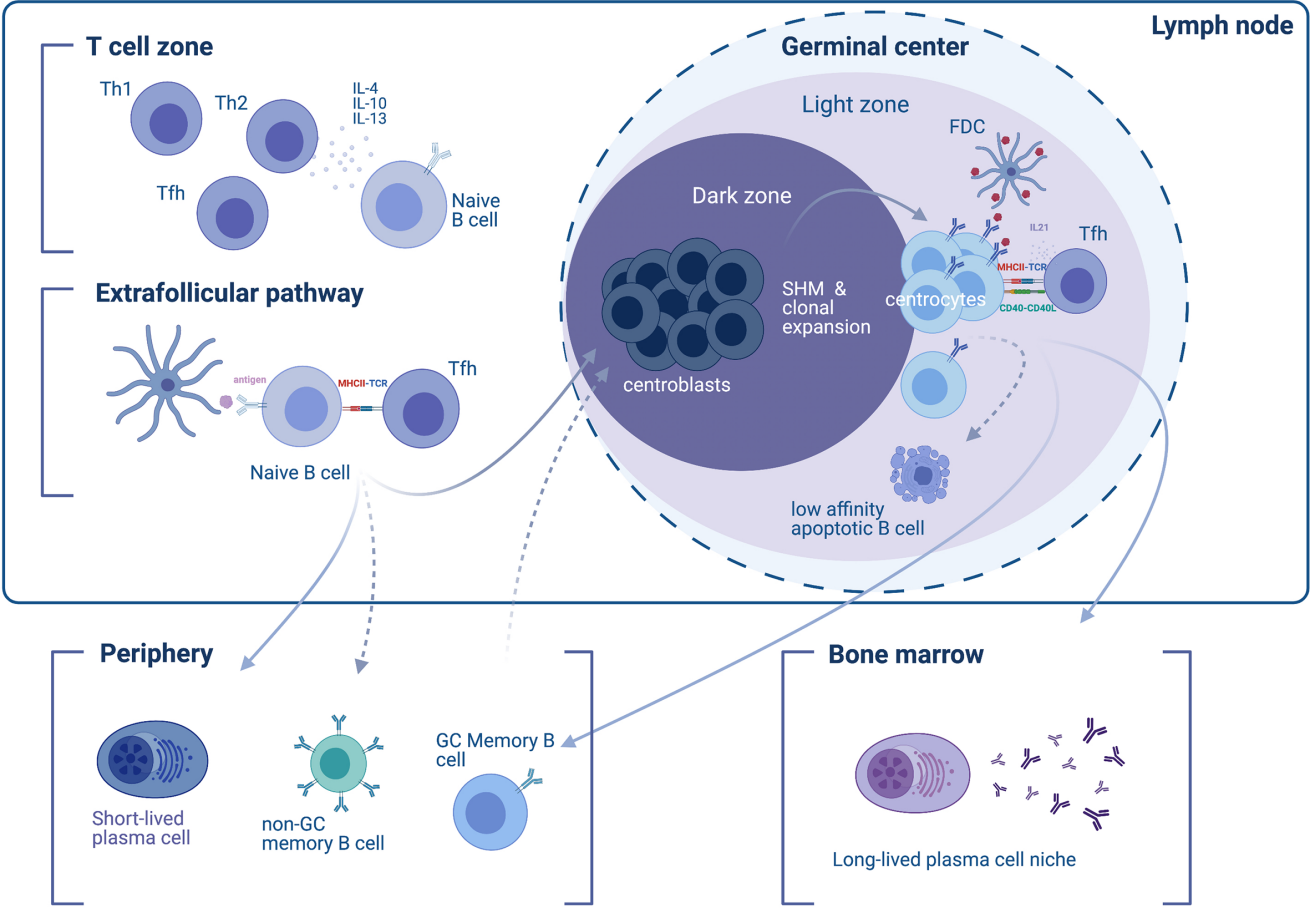
Differentiation of B cells into short- and long-lived antibody-secreting cells. In the initial phase of the immune response to a T cell-dependent antigen, responding naïve B cells appear in the T cell zone of the lymph node (upper left), where their development and differentiation is facilitated by T cell-secreted cytokines. T helper type 2 (Th2) type cytokine secretion, such as IL-4, -10, and -13 favors the induction of an IgG4 response. B cells enter the extrafollicular pathway and undergo B cell receptor (BCR) activation by encountering antigens on follicular dendritic cells (FDCs), which they then present to T follicular helper (Tfh) cells through MHC-II. The extrafollicular pathway gives rise to (i) short-lived plasmablasts (SLPBs) that enter the periphery, and (ii) germinal center (GC)-independent memory B cells. In a second phase, activated B cells enter the GC dark zone, where they mutate (a process called somatic hypermutation) and clonally expand (therefore termed centroblasts). B cells cycle between the dark and the light zone (where they are termed centrocytes). The dynamic cycle of the GC allows centrocytes that entered the light zone to be chosen based on the affinity of their BCRs to the antigen. Low-affinity B cells that are not presenting antigen on their BCRs will eventually become apoptotic and die. B cells that do present the antigen receive help from Tfh through CD40L and IL21 survival signals. The end-products of the GC reaction are (i) memory B cells, and (ii) long-lived plasma cells (LLPCs). GC memory B cells will enter the periphery and re-enter the GC upon BCR stimulation. LLPCs exit the GC and find a survival niche, typically the bone marrow.

Observations of follicular and extrafollicular responses rely mainly on rodent models. Shortly after exposure to a T-cell-dependent antigen, responding B cells and T cells appear at the B cell follicle/T cell zone border of the lymph node (26). The initial humoral response involves B cells differentiating into SLPBs with the help of T follicular helper cells (Tfh) at extrafollicular sites (2, 27) (**Figure 1**). These extrafollicular B-cell responses generate many of the early-induced antibodies approximately four days to one week after exposure to the antigen (2, 28). In canonical responses, the extrafollicular response reverts after the first week and gives its place to the GC responses. However, extrafollicular responses persist in non-canonical responses [e.g. in the setting of Salmonella (29) and Borrelia infection (30) or certain types of autoimmunity such as rheumatoid arthritis patients’ synovia (31) and lupus-prone mice (32)]. Although characteristics of the GC response (somatic hypermutation, class-switch recombination and generation of B cell memory) can also be found in canonical and non-canonical extrafollicular responses (29, 31–37), they are present to a lesser extent. One exception is the ability to establish LLPCs, which is absent in the extrafollicular response (38–41). Classical views regard the extrafollicular response as a response that is promiscuous and of low, yet detectable specificity (29, 36, 42).

### Germinal Center Responses

Development of B cell immunologic memory and differentiation of B cells into high-affinity LLPCs primarily occurs within secondary lymphoid tissue—specifically the lymphoid follicles (B cell follicles) of the lymph nodes or the spleen, which harbor structures known as GCs. After the initial B cell response, the initiation of GCs is orchestrated by various immune cells, including B cells, helper T cells (such as Tfh cells driven by bcl-6), follicular dendritic cells (FDCs), and macrophages, as well as cytokines such as IL-6 (43–46). Since the discovery of GCs as affinity-maturation entities (47), ﻿microscopy-visualized GC reactions (48, 49) have shed light onto GC structure and dynamics. Two zones comprise the GC: the light zone and the dark zone; the light zone facilitates interaction with the antigen *via* Tfh and FDCs in order to select higher-affinity B cell clones while the dark zone facilitates B cell proliferation and somatic hypermutation in order to generate candidates for clonal selection in the light zone (50) (**Figure 1**). Accordingly, dark-zone B cells (centroblasts) engage mitosis-related genes and activation-induced cytidine deaminase (AID), thus enabling somatic hypermutation and class-switch recombination (51). Mature GCs allow entry to naïve B cells in addition to re-entry of higher-affinity clones, as repeated involvement of memory B cells in GC reactions has been observed in human lymph nodes (52). As a consequence, exported LLPCs display higher affinity and more somatic mutations compared to memory B cells (53). Overall, higher-affinity clones that mature within the dark zone differentiate into either LLPCs ﻿that migrate to the bone marrow or memory B cells, while lower-affinity clones undergo apoptosis.

### A Continuum of Antibody-Secreting Cells

ASCs exiting extrafollicular or follicular maturation processes are grossly divided into SLPBs and LLPCs and are connected to specific immunophenotypes measured with flow cytometry. More specifically, SLPBs are often described as CD20^-^ CD19^med/+^ IgD^-^ CD27^hi^ CD38^hi^ (of those, some but not all are also CD138^+^), while LLPCs are described as CD20^-^ CD19^-^ CD38^hi^ CD138^+^ (54). It should be taken into account that as B cells differentiate toward high-affinity LLPCs, a phenotypic continuum is formed that does not entirely fit into the two immunophenotypes. Accordingly, a minority of presumably immature circulating SLPBs retain CD20 expression (55), and a minority of bone marrow LLPCs retain CD19 expression (56, 57). Both markers (CD20 and CD19) are ultimately lost as CD38 and CD138 expressions peak in Blimp-1-driven LLPCs.

### Mechanistic Differences Between IgG1 and IgG4 Responses

While IgG1 is the most predominant IgG subclass in healthy adults (more than 60% of total IgG) and IgG4 by far the rarest (approximately 5% of total IgG) (58–61), IgG4 is of special interest not only because it is related to autoimmunity but also because of its anti-inflammatory properties and its coexistence with Th2-driven IgE (allergic) responses (62). In beekeepers, chronic exposure to allergen leads to upregulation of both IgE and IgG4 (63); IgG4 competes with IgE for the same antigen but has weak effector functions [mobilization of complement-dependent cytotoxicity (CDC) and antibody-dependent cellular cytotoxicity (ADCC)]. Moreover, IgG4 cannot crosslink the antigen as it exchanges its Fab arms to become functionally monovalent (64). Consequently, production of antigen-specific IgG4 protects beekeepers from IgE-mediated allergy. Allergen tolerization therapy (e.g., grass pollen) is clinically effective and induces an IgG4 antigen-specific response. Of note, repetitive or prolonged antigenic stimulation seems to elicit an IgG4 response in other settings as well, such as in chronic biologic therapeutics administration (e.g. clotting factor VIII, natalizumab, adalimumab) (65–67), repeated immunization (68), and helminth infection (69–73).

Both IgG4 and IgE class-switch and production are induced by IL-4 and IL-13 (74–76), however additional IL-10 signals divert Ig production in favor of IgG4 (77–79). Allergic sensitization and immediate hypersensitivity points to the presence of IgE memory and total IgE titers persist in actively atopic patients (10, 62, 80). Data from murine models point to IgE B cells showing difficulty remaining in the GC and generating memory B cells and LLPCs (81, 82). This is corroborated by a post-seasonal total IgG4/IgE titer drop (62) and transient-only IgE increases seen in non-atopic children (83). IgG4 B cells seem to be equally ineffective in producing LLPCs. Compared to IgG1 B cells, human IgG4 B cells express less CXCR4, a chemokine important for bone marrow chemotaxis, and low numbers of IgG4 cells are observed in human secondary lymphoid organs (84). Data from IgG4 related disease (IgG4-RD) also support the notion that the generation of LLPCs is diminished in IgG4 responses as levels of circulating IgG4 SLPBs correlate with total IgG4 levels. In addition, rituximab treatment affects a significant drop in IgG4 (and IgE) levels (85), but the drop in IgG1 titers is not as pronounced (86). It should be noted though that as the response to rituximab treatment can be partial, some LLPCs most likely do exist.

Regulatory T cell (Treg) involvement may be different in IgG4 and IgG1 responses. Apart from extrafollicular Tregs that could control GC initiation, follicular regulatory T cells (Tfr) may balance Tfh cells and participate in determining the fate of B cells. Tfr cells may either directly suppress GC B cells through CTLA-4-mediated inhibition of CD80/CD86 co-stimulatory signaling or indirectly do so through IL-10 secretion acting on Tfh cells (87). Importantly, allergen-specific Tregs from healthy individuals can suppress IgE and induce IgG4 production *ex vivo via* IL-10 and TGF-β (88, 89). During human helminth infection (which causes IgE and IgG4 elevation), Tregs that produce IL-10 and inhibit effector T cells can be found in the peripheral blood and may play a role in limiting inflammation (90), while in murine models of helminth infection, Tregs expand, produce IL-10, and can limit the Th1 more than the Th2 response (91). Similarly, Tregs are expanded in the peripheral blood of IgG4-RD patients (along with IgG4 and Th2 cells) (92, 93) and infiltrate target organs (along with IgG4 cells) (94). Conversely, in MG mediated by predominantly IgG1 autoantibodies against AChR, patients have been shown to harbor dysfunctional Tregs (95), and further, induction of Tregs *via* GM-CSF effectively suppressed experimental autoimmune MG (96). In pemphigus vulgaris (PV) mediated by predominantly IgG4 autoantibodies against desmoglein, contradicting data that Tregs have both not been able to suppress (97) and have suppressed autoimmune responses (98) are reported. These results underline the need for further investigations into the role of Tregs in autoantibody-mediated autoimmunity.

### Class-Switch Recombination in the Setting of Allergy and Autoimmunity

**Figure 2:**
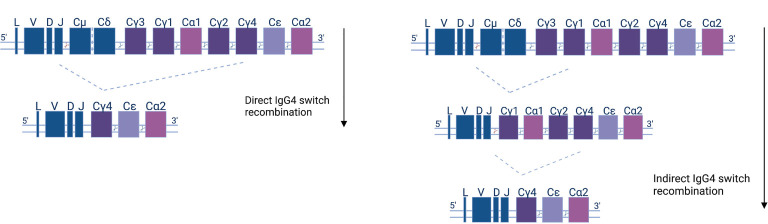
Human antibody isotype and class-switch recombination; diagram of the organization of the heavy chain gene locus. The constant genes are shown as squares and their width represents the relative gene size. Constant μ and δ (Cμ &Cδ) genes, preceded by the leader (L) and variable (V), diversity (D), or joining (J) gene regions, are expressed early in the B cell development. Recombination events (indicated as cutting sites) replace the Cμ and Cδ genes with other isotypes and subclasses (Cγ1-4, Cα1-2, and Cϵ) according to the depicted downstream order. IgG4 subclass antibodies can either be generated directly (left panel)—by IgM or IgD recombination and loss of the respective IGHC genes—or indirectly in two steps (right panel).

Class-switching recombination (CSR) is a fundamental change connected to the maturation of B cells as they evolve towards antibody secretion in response to antigens. CSR is facilitated by AID and materialized by the excision of DNA fragments and the subsequent joining of previously distant regions. As a consequence, CSR can only happen in the 5’-3’ direction of chromosomal topology of the different (corresponding to different classes and subclasses) constant region fragments (**Figure 2**). The use of high-throughput, next-generation sequencing (NGS) of the B cell receptor variable region and the beginning of the constant region (which can determine class and subclass) offers direct insight into human CSR mechanisms. Mutational analysis allows for the construction of B cell lineage trees, and at the same time, subclass is assigned to clonal family members. That way, one can pinpoint CSR with the use of somatic mutations as a ‘molecular clock’ (99). NGS analysis shows that the majority of naïve IgD/M switch to proximal classes (IgG3, IgG1, IgA1), and that the proximal classes then (secondarily, or indirectly) switch to more distal classes (IgG2, IgG4, IgA2). Indeed, more distal subclasses display greater mutational load, on average (61, 100–105).

CSR and origins of IgE and IgG4 have been extensively exanimated in allergy and autoimmunity. In allergy, whole-repertoire NGS data from healthy and allergic individuals indicate that indirect switching from IgG1 is the primary source of IgE, while indirect switching from IgG4 is also significant (given overall rarity of IgG4) (103, 106). In accordance, examination of antigen-specific clonal families in allergic individuals showed common presence of IgE with predominantly IgG1, and to a lesser degree, IgG4 B cells within the same clonal family (107, 108). Most importantly, in pemphigus vulgaris (PV), a bullous skin disease mediated by pathogenic autoantibodies (mostly IgG4) against desmoglein, the NGS approach showed that IgG4 antigen-specific cells are not predominantly formed from IgG1 (or IgA) precursors. In fact, IgG1 and IgG4 desmoglein responses evolve in parallel along the same lineage tree, and it is plausible that the IgM-to-IgG4 direct switch is predominant for antigen-specific cells (109).

### Mechanistic Differences Between IgG1 and IgG4 Antibodies

IgG1 and IgG4 autoantibodies bear different mechanisms of pathogenicity because of their molecular characteristics (e.g. P331S, L234F, P228S, A327G, and R409K amino acid substitutions in the IgG4 Fc region) (59, 79). Pro-inflammatory IgG1 autoantibodies activate CDC and ADCC through their constant fragment (Fc) and moreover can crosslink the antigen and affect its internalization. In contrast, IgG4 antibodies constitute a principally anti-inflammatory IgG subclass and have limited ability to mobilize CDC and ADCC due to a low affinity for C1q complement components and Fcγ receptors (110), however lgG4 antibodies can block the ligand-receptor interaction of the target antigen. One cardinal feature of IgG4 antibodies is that they undergo Fab-arm exchange, which results in bivalent binding properties of different specificities for each arm and thereby lose their ability to crosslink (64). This Fab-arm exchange effectively leads to functional monovalency of IgG4 antibodies. In addition to Fab-arm exchange, the lower mobility of IgG4s due to their shorter—compared to IgG1—hinge region complicates their structure, making IgG4 even less likely to be crosslinking antigens (111). Overall, the IgG4 subclass remains relatively poorly studied yet plays a fundamental role in neurological and non-neurological autoimmunity (58–61).

## Autoimmune Neurological Diseases Mediated by IgG1 and IgG4 Autoantibodies

In many neurologic diseases, some of them newly defined, pathology is mediated by autoantibodies against surface/extracellular protein antigens. Among those are MG, chronic inflammatory demyelinating polyradiculoneuropathy (CIDP), neuromyelitis optica and neuromyelitis optica spectrum disorders (NMOSD), acute disseminated encephalomyelitis (ADEM), relapsing optic neuritis (ON), pediatric acquired demyelinating syndromes, and autoimmune encephalitis (AE) (112–116). Such neuronal autoantibodies can affect multiple facets of the CNS and PNS as they target a wide spectrum of antigens, neurotransmitter receptors, ion channels and glycoproteins (**Table 1**). Clinical manifestations of neurological diseases mediated by autoantibodies are diverse and include fatigability, weakness, sensory and visual disturbances, movement and sleep disorders, epileptic seizures, decreased consciousness, and various cognitive symptoms. Serum and CSF autoantibodies have transitioned from being mere diagnostic tools to being defining factors of the clinical spectrum; and moreover, autoantibody titers can sometimes serve as markers of disease activity and more often of response to treatment. Of note, neuronal autoantigens can be classified into two categories depending on their localization: surface or intracellular. Pathogenicity of the latter is questionable, and as accessibility of intracellular targets to autoantibodies is limited in intact cells, the humoral response could be secondary to a primary cellular-damage event. In these cases, the primary response could involve a T cell (162, 212) or other cytotoxic immune cell-mediated mechanism. Here, we focus on cell surface protein antigens, where humoral responses are directly implicated in disease pathology.

**Table 1:** Autoimmune neurological diseases mediated by IgG1 and IgG4 autoantibodies.

Disorder	Subclass	Prevalence	Antibody localization	HLA restriction	Complement involvement
MuSK MG	IgG4 (117–119)	1.9-2.9/1.000.000 (120)	Serum (118, 119, 121)	HLA-DR14-DQ5 haplotype (122, 123)	N/A
AChR MG	IgG1 & IgG3 (124–126)	4.3-18/1.000.000 (120)	Serum (124, 125, 127, 128)	No association (but DQB1*05:02 and DRB1*16 (129)	Yes (126, 130–132)
AQP4 NMO(SD)	IgG1 (133)	27-100% of 0.7-1.09/100,000 (134, 135)	Serum, CSF (136–138)	HLA-DRB1*03:01 (139, 140)	Yes (141–143)
MOG MOGAD	mostly IgG1, also IgG2, IgG3 (144, 145)	1.9/100,000 (146)	Serum, CSF (145, 147, 148)	No association (149)	Yes (150, 151)
NMDAR Encephalitis	IgG1 and IgG3 (152–157)	0.6/100.000 (146)	Serum (158)	Weak association with the HLA-B*07:02 allele (159)	No (153, 160–162)
IgLON5 disease	mostly IgG4, also IgG1 (163). All 4 IgG subclasses detected (164)	1/150.000 (164, 165)	Serum and very frequently in the CSF (164)	HLA-DRB1 & HLA-DQB1 (163)	No (166)
LGI1 Encephalitis	IgG4 (167, 168)	0.7/100.000 (146)	Serum, in 80–90% in CSF (169, 170)	HLA-DRB1*07:01–DQB1*02:02 (171–173) & HLA-DR7 and HLA-DRB4 (174)	Limited evidence (162)
CASPR1 CIDP	IgG4, IgG1, IgG3 (175), IgG2/3 (176)	1.9-4.3% of CIDP: 0.7-10.3/100.000 (177, 178)	Serum (175)	N/A	Limited evidence (179)
Contactin1 CIDP	IgG4 (175, 180)	0.8% of CIDP: 0.7-10.3/100,000 (175, 177)	Serum (175)	All CIDP HLA-DR3 & DR3/DQ2 (181)	Limited evidence (182)
Neurofascin CIDP (NF155 & NF186)	IgG4 (175, 180, 183), IgG1, IgG3 (175)	2.9-7% of CIDP: 0.7-10.3/100.000 (175, 177, 184)	Serum (184)	HLA-DRB15 (185) HLA-DRB1-15 & DQB1 (186)	No (184)
GABA-A Encephalitis	IgG1 (94%) or IgG3 (6%) (187)	~50 cases (187, 188)	Serum, CSF (189, 190)	N/A	N/A
GABA-B Encephalitis	IgG1 (191)	~63 cases (191–193)	Serum, CSF; in 25% high titer in CSF (192)	No association (173)	Yes (194)
DPPX Encephalitis	IgG1 and IgG4 (195)	<40 cases (195–198); <1/1.000.000 (ORPHA:329341)	Serum, CSF	N/A	N/A
mGluR5 Encephalitis	IgG1, IgG1/IgG2, IgG1/IgG3 (199)	~20 cases (199, 200)	Serum, CSF (199)	N/A	Not likely (199)
CASPR2 Encephalitis	IgG4 (167, 170)	~100 cases (170, 201, 202)	Serum, CSF (203)	HLA-DRB1*11:01-DQA1*05:01-DQB1*03:01 (171)	Limited evidence (204, 205)
GlyR Encephalitis	IgG1 and IgG3 (152–157)	~100 cases (155, 206)	Serum, CSF (155, 158, 207, 208)	N/A	Yes (155)
AMPAR Encephalitis, (GluA1 & GluA2)	IgG1 (209)	<100 patients (210, 211)	Serum, CSF (193)	N/A	Limited evidence (209)

MG, myasthenia gravis; CIDP, chronic inflammatory demyelinating polyradiculoneuropathy; NMOSD, neuromyelitis optica spectrum disorder; MOGAD, myelin-oligodendrocyte glycoprotein antibody disorder; AE, autoimmune encephalitis; N/A, not available.

In addition to IgG1 autoantibodies, several different CNS or PNS antigens have been identified as targets of IgG4 autoantibodies, and this discovery has enabled us to categorize autoimmune diseases by pathophysiological mechanism. IgG4 autoantibody-mediated diseases are not driven by antibody effector functions but by direct and mechanical disruption of ligand-receptor interactions (175). Examples include antibodies specific to MuSK, a protein that is instrumental in the agrin-LRP4 pathway leading to AChR clustering (213); LGI1, a secreted protein that stabilizes the transsynaptic complex between the pre and postsynaptic receptors, ADAM23 and ADAM22 (169, 214, 215); and the protein complex of contactin-1, neurofascin-155 and caspr-1, which anchors myelin loops to the axon at the Ranvier paranode (216). A further interesting aspect of autoantibody-mediated neurological diseases is HLA restriction seen in patients as compared to healthy controls, likely meaning that specific antigenic peptides are better presented to T cells by specific HLA alleles (217, 218). This is relevant to B cell function, as B cells can pick up antigens with the B cell receptor (BCR) and process them and effectively present them *via* MHC II to T cells (219). Such a restriction has been shown both in diseases mediated by IgG1 and by IgG4 autoantibodies, and strong associations to specific—mainly class II—HLA haplotypes have been detected, with odds ratios exceeding 8 in disorders such as encephalitis with autoantibodies against leucine-rich glioma-inactivated protein 1 (LGI1) and contactin-associated protein 2 (caspr2) (171), chronic inflammatory demyelinating polyradiculoneuropathy (CIDP) with autoantibodies against neurofascin 155 (NF155) (185) and IgLON5 disease (220).

## Evidence on the Existence of Long- and Short-Lived ASCs in Neurological Autoimmunity

The short- and long-lived nature of pathogenic autoantibodies is directly relevant to immunotherapeutic strategies. There are indications from the study of MG—the prototype of autoantibody-mediated diseases—that there is a systemic difference between predominantly IgG1 and predominantly IgG4 autoantibody responses regarding the longevity of ASCs. We have therefore examined the relevant evidence in the more common neurologic autoantibody-mediated disease entities. Data can be divided into two categories. First, we examined autoantibody titer response to rituximab, the CD20^+^ B cell depleting monoclonal antibody that has revolutionized neurologic therapeutics, and a rapid decline in autoantibody titer post-rituximab supports the short-lived nature of ASCs. Second, we reviewed studies that have directly examined ASCs by employing an array of techniques, from immunohistochemistry and bulk B cell culture to single B cell cloning and production of monoclonal antibodies. In particular, immune phenotypes of B cells from which antigen-specific monoclonal antibodies are derived provide information about the short- or long-lived nature of ASCs. These two groups of data are summarized in **Table 2**.

**Table 2:** Summary of data supporting presence of short and long-lived ASCs in neurological disorders.

Antigen	Subclass	Post rituximab antibody titers	Findings regarding short- and long-lived antigen-specific ASCs	SLPB presence	GC/LLPC presence
AChR	IgG1	No change or mild decrease (9, 11, 13, 14)	Cultured BM, thymus, and lymph node cells produce AChR Ab (221); GCs present in thymus (222); AChR-specific B cells present in thymus (223, 224) are HLA-DR^low^ plasma cells (225)	no	yes
MuSK	IgG4	Marked decrease (6, 7, 9, 20, 213)	SLPBs produce MuSK Ab (213, 226)	yes	no
AQP4	IgG1	Significant decrease and no change both reported (227–230)	CD20^+^ B cells and CD138^+^ (SLPBs or LLPCs) cells found in CNS (231, 232); Peripheral blood SLPBs increased in relapses; SLPBs produce AQP4 Ab in culture (233) [could not be reproduced from frozen cells (234)]; presence of AQP4-specific CD138^+^ cells (SLPBs or LLPCs) in CSF (235)	yes	possible
MOG	IgG1	Decrease in MFI, yet MOG-Ab remained detectable (236)	CD20^+^ B cells found in CNS (237); Peripheral blood PBs not increased in relapses (238)	no	no
NMDAR	IgG1	Marked decrease in one patient (239)	B cells and CD138^+^ cells found in CNS (160, 162, 240, 241); Peripheral blood SLPBs increased in one patient (239); GC-like structures, SLPBs and CD20^-^ CD138^+^ LLPCs found in teratomas, and teratoma lymphocytes produce NMDAR Ab (153, 242); NMDAR-specific SLPBs or LLPCs found in CSF (241, 243)	yes	yes
IgLON5	IgG4, IgG1	N/A	Few CD20^+^ B cell in brain (166, 244, 245)	no	no
LGI1	IgG4	Marked decrease in 5/6 patients (246, 247)	LGI1-specific CD138^+^ cells (SLPBs or LLPCs) found in CSF (248)	possible	possible
Contactin1	IgG4	Marked decrease (249)	N/A	no	no
NF155	IgG4	Marked decrease (249, 250)	N/A	no	no
DPPX	IgG4, IgG1	Decrease (251)	N/A	no	no
Caspr2	IgG4	Marked decrease (252)	N/A	no	no
mGluR5	IgG1, IgG4	Decrease (253)	N/A	no	no
GABA-B	IgG1	N/A	CD19^+^ CD138^+^ SLPBs found in CSF; CD138^+^ cells found in brain parenchyma (254)	yes	possible

ASCs, antibody-secreting cells; AChR, acetylcholine receptor; BM, bone marrow; GC, germinal center; MuSK, muscle-specific kinase; NF155, neurofascin155; AQP4, aquaporin 4; SLPBs, short-lived plasmablasts; LLPCs, long-lived plasma cells; MFI, mean fluorescence intensity on flow cytometry cell-based assay; MOG, myelin oligodendrocyte glycoprotein; NMDAR, N‐methyl‐d‐asparate receptor; LGI1, Leucine-rich glioma-inactivated protein 1; DPPX, dipeptidyl-peptidase-like protein-6; Caspr2, contactin-associated glycoprotein2; mGluR5, metabotropic glutamate receptor 5; GABA-B, gamma aminobutyric acid receptor B; N/A, not available.

It should be noted that rituximab-induced B cell depletion is materialized by ADCC, CDC, and antibody-dependent cellular phagocytosis (ADCP) (255). Circulating CD20^+^ B cells become undetectable almost immediately after rituximab treatment, and levels remain low for at least 6 months. Repeated dosing every 6 months can affect longer (than 6 months) B cell depletion after treatment ends (256–259). Moreover, rituximab affects a reduction but not a compete depletion in B cells of lymph nodes (260). Finally, repeated rituximab dosing results in gradual total serum IgM and IgG reduction (261).

## Myasthenia Gravis: The Prototype

Evidence from several case series suggests that rituximab is clinically effective in the majority of MG patients; especially in patients with MuSK MG the improvement is more pronounced compared to AChR MG (8, 9, 13, 16–24). Despite the many reports of good efficacy of rituximab in AChR MG, a one-year phase 2 trial of rituximab (NCT02110706) did not meet the primary endpoint, which was achievement of a 75% reduction in mean daily prednisone. This was possibly due to the study duration and a high percentage of patients meeting the endpoint in the placebo arm. The secondary endpoints, improvement in quantitative MG scales, was greater in the rituximab arm but the difference was not significant. This clinical difference between MuSK and AChR MG is reflected in autoantibody titers post-rituximab. Most patients with MuSK MG receiving rituximab show marked decline in MuSK autoantibody titer (7–9, 20). In contrast, titer decline in AChR MG is variable and in many patients is less pronounced (9, 11–14). Interestingly, the intensity of rituximab induction seems to be proportionate to the durability of the response of MuSK MG patients (262).

In parallel, cellular approaches have also contributed to deciphering of the short- or long-lived nature of ASCs in MG. In AChR MG, cultured bone marrow cells produced higher concentrations of AChR autoantibodies compared to peripheral blood, thymus, and lymph node lymphocytes, thus providing direct evidence for LLPC involvement in autoantibody production (221). Moreover, the presence of GCs in the thymus of early onset AChR MG patients underscores the ability to produce LLPCs (222). Of note, such thymic hyperplasia is not present in MuSK MG (263). The ability of thymic cells to produce AChR autoantibodies is well documented with different approaches (221, 223–225) and the contribution of LLPCs to this production is based on indirect observations, such as the absence of HLA-DR expression of some ASCs (225). Thymic ASCs can potentially survive in the thymus through constitutive stimulation by autoreactive T cells, and AChR-specific T cells have been found in the periphery of MG patients (264). In MuSK MG, evidence points to the presence of antigen-specific ASCs within the peripheral SLPB compartment. First, cultures of CD3^-^ CD14^-^ CD19^med/+^ IgD^-^ CD27^hi^ CD38^hi^ SLPBs from the peripheral blood of MuSK MG patients produced MuSK autoantibodies (226). Second, IgG4 and IgG3 MuSK-specific monoclonal autoantibodies were able to be produced from the CD3^-^ CD14^-^ CD19^med/+^ IgD^-^ CD27^hi^ CD38^hi^ SLPB fraction with the use of complementary single-cell sorting strategies (213, 226, 265). While this evidence for the presence of MuSK-specific SLPBs in MuSK MG is strong, the presence of MuSK-specific LLPCs cannot be excluded. Taken together, data point to autoantibody production that relies more on SLPBs in the predominantly IgG4 MuSK MG than in the predominantly IgG1 AChR MG, where the presence of antigen-specific LLPCs is well documented.

## Aquaporin-4 Neuromyelitis Optica Spectrum Disorder

In NMO and NMOSD with IgG1 autoantibodies against aquaporin-4, rituximab has demonstrated remarkable clinical efficacy (229, 230). Titer response to B cell depletion with rituximab, however, has been variable. In a report about three patients, rituximab infusions led to parallel decreases in CD19^+^ count and AQP4 autoantibody titer. The response did not lead to total eradication of autoantibodies and was not particularly prolonged, since titers increased again along with CD19^+^ B cells after approximately one to one-and-a-half years later (227). This pattern was corroborated in a single patient where AQP4 antibodies were tested in nine different centers for validation purposes (266). In subsequent reports, titers for seven to thirteen patients were reported as a function of time, and responses to rituximab were mixed: autoantibody titers were refractory to rituximab in some patients, dropping in response to rituximab in others, and autoantibodies were beneath the level of detection in a third group (229, 230). A further study of the titers of five AQP4^+^ patients demonstrated a transient and incomplete response of titers to rituximab in three patients and a complete lack of response in the remaining two (228). Collectively these data point to variable titer responses and therefore to the presence of both AQP4-specific SLPBs and LLPCs.

Cellular approaches, both histopathological and *ex vivo* bulk and single-cell, have added important pieces of information on the nature of ASCs. First, biopsy and autopsy CNS studies have shown (i) perivascular B cells to a varying degree in addition to T cells (231, 267, 268); (ii) perivascular CD138^+^ cell infiltrates in one patient (232); and (iii) IL-6 transcripts in another patient (269). Second, peripheral blood CD19^+^ CD27^hi^ CD38^hi^ SLPBs were shown to increase in AQP4 autoantibody-positive NMO patients, more so during relapses. Importantly, these cells were able to produce AQP4 autoantibodies when cultured in the presence of IL-6, a cytokine also known to be increased in NMO relapses (270). These results were not reproducible in a follow up study, but it employed frozen cells, which could have impacted the viability of ASCs (234). Third, in a study of single CSF B cells from an early NMO patient, 3.7% of the CSF lymphocyte population was CD19^+^ CD138^–^ B cells, and 0.9% were CD138^+^ ASCs; most of the CD138^+^ CSF ASCs (70.5%) were CD19^+^ CD138^+^ SLPBs, the rest were CD138^+.^CD19^-^ LLPCs. Production of monoclonal antibodies from these mostly IgG1 (and rarely IgG2) CD138^+^ SLPBs or LLPCs demonstrated AQP4 specificity and somatic mutations (235). Moreover, AQP4 CSF SLPBs were found to be clonally related to peripheral plasmablasts as well as peripheral memory cells (271, 272). Of note, the involvement of IL-6, a cytokine that promotes GC formation as well as ASC survival, is further supported by the use of the anti-IL-6 receptor antibody tocilizumab in rituximab-resistant, aggressive cases of NMO (273). Interestingly, treatment of an AQP4 NMO patient with tocilizumab led to reduction in the frequency of CD19^int^ CD27^hi^ CD38^hi^ SLPBs and anti-AQP4 antibody titer within one month of treatment (274). Taken together, data from serological and cellular approaches support a role for both short- and long-lived ASCs in AQP4 NMOSD pathology.

## Myelin-Oligodendrocyte Glycoprotein Antibody Disease

The pathological roles of anti-MOG IgG1 antibodies are not fully understood, and some MOG antibody disease (MOGAD) patients exhibit high titers of autoantibodies with pathogenic properties, while other patients—along with healthy and disease controls—exhibit lower titers (275, 276). Of note and in contrast to AQP4 NMOSD, clinical response of MOGAD patients to rituximab is modest, with up to a third of patients relapsing despite full depletion of peripheral B cells (236, 277–279). Currently there are no reliable predictors of inadequate response to rituximab in MOGAD patients, and the post-rituximab pattern of memory B-cell compartment population did not differ between responders and non-responders (236). In a case series of 16 MOGAD patients who received rituximab, mean fluorescence intensity (MFI) in the flow cytometric autoantibody detection cell-based assay decreased in most patients, while MOG antibodies remained detectable, which suggests a role for both SLPBs and LLPCs in MOG-IgG production (236). Of note, MFI could be viewed as a correlate of autoantibody titer. In pathological investigations, CD20^+^ B cells have been identified in the brain of MOGAD patients (237). Moreover, unlike patients with AQP4-NMOSD, where SLPBs were elevated, peripheral blood SLPBs were not elevated in the active phase of MOGAD patients (238). Taken together, serological and cellular data point to the presence of a mixed (both short- and long-lived) population of MOG autoantibody-secreting cells.

## NMDAR Encephalitis

Antibodies against the N‐methyl‐d‐aspartate receptor (NMDAR) are predominately of the IgG1 subclass (280). Patients with NMDAR encephalitis generally respond to rituximab, and in one case titers were undetectable post-treatment (152, 239, 280–282). In an effort to enhance the effect of rituximab induction treatment, two non-randomized trials observed clinical benefit from repeated monthly dosing of rituximab in addition to induction dosing (283); and the addition of tocilizumab to rituximab (240). Several investigations of cellular immunopathology have been applied in NMDAR encephalitis. First, a histopathological study of autopsy and biopsy material revealed the presence of B cells and CD138^+^ cells in perivascular regions and interstitial spaces that could provide a local source of antibody production, as well as the presence of T cells and the absence of complement deposits and neuronal loss (153, 160, 162, 241). Moreover, a histopathological study of teratomas (seen in 20% of patients with NMDAR encephalitis) has shown GC-like structures harboring CD3^+^ T cells, CD20^+^ B cells, CD19^+^ CD27^hi^ CD38^hi^ SLPBs, and CD20^-^ CD138^+^ plasma cells (284, 285). Importantly, NR1 NMDAR subunit expression was high in teratoma B cells, and teratoma-derived lymphocytes were able to produce NMDAR autoantibodies when stimulated in culture.

Two studies analyzed the intrathecal cellular response in NMDAR encephalitis with the use of flow cytometric cell sorting and construction of monoclonal antibodies from single cells (241, 243). Cells that were NMDAR-specific were identified as IgG3 CD3^-^ CD14^-^ CD16^-^ CD20^+^ IgD^-^ CD27^+^ memory B cells, IgG1 and IgG2 CD3^-^ CD14^-^ CD16^-^ CD27^+^ CD38^+^ ASCs (could be both SLPBs or LLPCs) (243) and CD19^+^ CD138^+^ SLPBs (241). NMDA specificity was associated in most, but not all, cases with somatic mutations, which indicates some degree of affinity maturation. Interestingly, CD19^+^ CD138^+^ SLPBs disappeared from the CSF after immunotherapy with methylprednisolone, mycophenolate and azathioprine (241). These collective data suggest involvement of IgG1, IgG2, and IgG3, both SLPBs and LLPCs, in the production of the pathogenic NMDAR autoantibodies.

## IgLON5 Disease

In IgLON5 disease, patients harbor autoantibodies of all four IgG subclasses, with some studies reporting a predominance of IgG4 and others of both IgG1 and IgG4 (163, 164, 286–288). *In vitro* experiments have shown that IgG1 (not IgG4) antibody binding to IgLON5 results in protein internalization and an overall decrease of neuron surface IgLON5 levels. This was not reversed when IgLON5 antibodies were removed, thereby suggesting permanent destruction of the protein’s biological function (286). In accordance, clinical data demonstrate better effectiveness of early compared to late immunotherapy (164, 288–290). Different series report rituximab use in 5–80% of IgLON5 patients (163–165, 288, 289, 291), and the response rate was calculated by a recent meta-analysis at 37.5% (292). More insight is needed about whether this variable response to rituximab is associated to the depletion of certain subclasses and whether prompt administration positively impacts patient outcome. In isolated cases where brain pathology was performed, few brain-infiltrating B cells were detected and no CD138 staining was reported (166, 244, 245).

## Autoimmune Encephalitis With LGI1 Autoantibodies

Leucine-rich glioma-inactivated protein 1 (LGI1) antibodies are mainly of IgG4 subclass (168). A study of rituximab treatment in six patients with LGI1 encephalitis resulted in clear improvement in only two patients. However, the treatment might have been applied too late in the course of the disease in refractory cases (247, 293). The response of autoantibody titers though was a marked reduction in all cases but one, where the decline was mild. These data are in agreement with titer responses in other IgG4 autoantibody-mediated disorders. Immunopathology studies in brain samples from human patients and cats with LGI1 encephalitis indicate participation of CD20^+^ B cells in CNS inflammatory infiltrates, as well as marked IgG and complement deposition (162, 294). The presence of complement deposition points to the putative role of other, coexisting, non-IgG4 antibodies as complement activating factors, or alternatively, points to the ability of IgG4 antibodies to mobilize complement despite classical views, possibly *via* altered IgG4 glycosylation and the lectin pathway (295–299), or IgG4 aggregation (79).

Single-cell approaches from the CSF of patients with long-lasting progressive LGI1 encephalitis identified IgG1, IgG2, and IgG4, LGI1-specific CD3^-^ CD14^-^ CD16^-^ CD20^+^ CD27^+^ memory B cells and CD3^-^ CD14^-^ CD16^-^ CD138^+^ ASCs (could be both SLPBs or LLPCs); V gene sequences of the LGI1-specific B cells were mutated (248). In a separate study, application of BCR NGS on both sides of the blood-brain barrier provided strong evidence in favor of GC reactions within the CNS. However, this was not shown for LGI1-specific B cells (300). Taken together, the serological evidence points to a predominance of short-lived LGI1 autoantibody-producing cells over LLPCs, while cellular data are not conclusive for the presence of a particular cell type.

### Chronic inflammatory demyelinating polyradiculoneuropathy

In CIDP with predominantly IgG4 autoantibodies against the paranodal components neurofascin-155 (NF155), contactin-1, or contactin-associated protein 1 (Caspr1), rituximab has been applied to corticosteroid and IVIg-refractory cases (249). Albeit limited by the low N given the rarity of the disease, antibody titers dropped significantly and rapidly in two patients (one with NF155 and one with contactin1 autoantibodies) after rituximab treatment, which correlated with marked clinical improvement. In a third patient (with NF155 autoantibodies), titers dropped but remained high, and a second rituximab infusion was required, after which autoantibodies were undetectable. In a larger cohort of 13 patients (8 with NF155 and 5 with contactin1 antibodies), rituximab administration resulted in a dramatic reduction of autoantibody titer that correlated with clinical improvement (250). One further study reported on titer drop (from 1:32,000 to 1:4,000) and clinical improvement after rituximab administration in a NF155 patient, even though the titers remained high (301). These studies provide evidence for a similar pattern of response in autoantibody-mediated CIDP that resembles MuSK MG.

## Other Autoantibody-Mediated Encephalitis Syndromes

In progressive encephalopathy with rigidity and myoclonus (PERM), where IgG1 autoantibodies against the glycine receptor have been found, rituximab has been associated with some improvement and lack of relapse (155, 302). In encephalitis with predominantly IgG1 autoantibodies against the GABA-B receptor (191), both partial and full response to rituximab have been noted in two patients (303). In encephalitis with predominantly IgG1 autoantibodies against the GABA-A receptor (187), rituximab has been administered (189), and a range of responses from full recovery to death has been documented (188, 304). Of interest, in a study of three patients with GABA-B encephalitis, CD19^+^ CD138^+^ SLPBs were seen in the CSF, along with CD138^+^ cells (that could be SLPBs or LLPCs) perivascularly in the brain parenchyma (254).

In dipeptidyl-peptidase-like protein-6 (DPPX) encephalitis, autoantibodies are of both the IgG1 and IgG4 subclass, but pathogenicity might be linked to IgG1 and its cross-linking functions (195, 196). In contactin-associated protein-2 (caspr2) encephalitis, autoantibodies are predominantly of the IgG4 subclass (170). In a metabotropic glutamate receptor type 5 (mGluR) encephalitis case series of 11 patients (199), autoantibodies were predominantly IgG1, but the co-existence of IgG1 with IgG4 autoantibodies has been noted in one case (253). Autoantibody titer data in response to rituximab treatment are available in all three disorders. In DPPX encephalitis, a significant clinical response to rituximab (195, 305) along with titer reduction (251) has been observed. In caspr2 encephalitis, a dramatic improvement along with autoantibody elimination has been observed (252), however post rituximab relapse has also been noted (306). Favorable clinical response to rituximab in Caspr2 encephalitis has further been observed in larger case series (170, 307). In mGluR encephalitis, rituximab has been associated with an improved course in two patients with IgG1 and IgG3 autoantibodies (199), with relapse upon rituximab discontinuation in a patient where the subclass was not specified (308), and with significant improvement along with titer reduction in a patient who originally harbored IgG1 and IgG4 autoantibodies (253). Overall, both clinical and titer responses seem to be more favorable in the case of IgG4 autoantibodies as in the case of Caspr2, and good responses in cases with IgG1 and mixed subclass autoantibodies have also been frequently noted; however, the limited number of patients given the rarity of the syndromes makes general conclusions difficult to draw.

## Non-Neurological Autoimmune Disorders Associated With Igg4 Autoantibodies

The paradigm we discuss for neurological diseases associated with or mediated by predominantly IgG4 autoantibodies is not confined to the nervous system. CD20^+^ B cell depletion therapy has been applied in a plethora of non-neurological diseases associated with IgG4 autoantibodies such as pemphigus vulgaris (PV), membranous nephropathy (MN), and thrombotic thrombocytopenic purpura (TTP). In all these diseases, B cell depletion has resulted in marked clinical improvement and rapid decrease in autoantibody titers, similar to IgG4 autoantibody-mediated neurological disorders (309–313). A specific mention should be given to PV, which was the first autoimmune disease described as predominantly IgG4 autoantibody-mediated and has been extensively studied. High quality data from a prospective, multicenter, open-label, randomized trial of continued rituximab administration in PV demonstrated complete and sustained remission at the end of the second year of follow-up in 89% of 46 patients who received rituximab; rapid normalization of anti-desmoglein-3 (DSG-3) antibody titers post B cell depletion was also shown, thereby underscoring the short-lived nature of ASCs producing anti-DSG-3 autoantibodies (314, 315). In TTP, rituximab induction therapy of 40 patients resulted in a rapid and sustained recovery of platelet counts and, in parallel, a rapid and sustained decrease of pathogenic, predominantly IgG4 anti- A disintegrin and metalloproteinase with thromboSpondin‐1 motifs; 13th member of the family (ADAMTS13) autoantibody titers (310). In membranous nephropathy, rituximab administration resulted in significant decline or disappearance of the predominantly IgG4 (likely pathogenic) autoantibodies against phospholipase A2 receptor in 68% of 35 patients within 12 months, correlating with partial or complete clinical remission (311). Taken together, these studies show that the short-lived nature of autoantibody-secreting cells (as evidenced by post-rituximab autoantibody titer data) in diseases associated with IgG4 autoantibodies is not a phenomenon restricted to the nervous system.

## Discussion

When collectively examining the titer of pathogenic autoantibodies against extracellular antigens (or correlates of an antibody titer on a flow cytometric cell-based assay like MFI), one can conclude that in disorders where IgG4 autoantibodies are prevalent, there is a marked decrease post-rituximab administration. This is the case in MuSK MG, in CIDP with antibodies against NF155 or contactin1, and in autoimmune encephalitis with LGI1 or Caspr2 antibodies, and also extends beyond the nervous system to disorders such as PV, TTP and MN. This is a clear indication that in these diseases, autoantigen-specific ASCs are short-lived. Further, decreases in post-rituximab titers are seen in disorders where IgG1 and IgG4 autoantibodies coexist, such as DPPX and mGluR5 encephalitis. In disorders where IgG1 autoantibodies are prevalent, rituximab treatment affects a variable titer response, meaning that in some patients, titers are refractory, in some patients, titers mildly decline, and in other patients the reduction of titer or MFI is more pronounced. This variable response is noted in both AChR MG and AQP4 NMO and NMOSD, but in MOGAD MFI decrease seems to be consistent. These results point to the presence of antigen-specific LLPCs in a significant number of patients harboring IgG1 autoantibodies, but also the presence of SLPBs. It should be noted that these studies are complicated by the fact that the disorders in question are rare and therefore the N is low. Moreover, titers are not systematically recorded pre- and post-rituximab. As titers offer valuable information about treatment responses, every effort should be made to record titers more frequently.

When collectively interpreting data from experiments that more directly examine autoantigen-specific ASCs, one can conclude that in disorders with IgG4 autoantibodies, the presence of LLPCs is not definitively shown. In MuSK MG, peripheral blood antigen-specific ASCs have been shown to have a SLPB phenotype, whereas in LGI1 encephalitis, CSF antigen-specific ASCs can express CD138. However, it was not specified whether these cells retain CD19 expression. Therefore, these cells could be either SLPBs or LLPCs, which means that the presence of some LLPCs in IgG4-mediated disorders cannot be excluded. In contrast, in diseases with IgG1 autoantibodies there is more definitive evidence for the presence of LLPCs. In AChR MG, production of autoantibodies from bone marrow cells and the presence of thymic GCs and HLA-DR^low^ antigen-specific cells all point to the existence and ability to generate autoantigen-specific LLPCs. In NMDAR encephalitis, the presence of GC-like structures and CD20^-^ CD138^+^ ASCs in ovarian teratomas also point to the existence of and ability to produce LLPCs. Moreover, CD138^+^ ASCs have been observed in the CNS and the CSF of AQP4 NMOSD, NMDAR and GABA-B encephalitis patients, and in the case of AQP4 and NMDAR, the antigen specificity of the CSF ASCs was demonstrated. Unfortunately, and similarly to the case of the IgG4 LGI1 encephalitis, a clear absence of CD19/CD20 staining and negativity prevents the definitive characterization of these cells as LLPCs. In such instances, use of an additional flow cytometric marker in combination with index sorting and the use of an additional immunohistochemical stain would permit capture of this mechanistically valuable piece of information.

In conclusion, it seems that the paradigm of the predominantly IgG4 MuSK and predominantly IgG1 AChR MG can be extrapolated to other autoimmune neurological (and non-neurological) disorders. Moreover, in disorders with IgG1 autoantibodies, the generation of antigen-specific LLPCs seems to occur to a greater extent as compared to IgG4 disorders. It should be noted, however, that a significant degree of variability exists and that both antigen-specific LLPCs can be generated in some patients with IgG4 autoantibody-mediated disorders, and SLPBs—perhaps more frequently—can be significant producers of autoantibodies in some patients with IgG1 disorders. Variability in relation to the nature of autoantibody-producing cells could also occur at different times in the same patient. This variability underscores the need for personalized medical approaches. Exceptions aside, a generally reduced ability to establish LLPCs in IgG4 responses is strongly supported by immunological observations on the longevity of ACSs from the field of allergy (both in humans and animal models) and IgG4-related disease. It could be the case that IgG4 autoantibody-mediated autoimmunity constitutes a mainly extrafollicular response, but follicular hyperplasia (in the absence of pathogenic antigen-specificity) has been observed in IgG4-RD (316). The tendency to generate predominantly IgG1 or IgG4 autoimmune responses may stem from HLA and/or non-HLA genetic differences (317, 318), but incomplete GWAS data (due to disease rarity) would have to be complemented by functional studies to better support such an argument. On the other hand, many aspects of IgG1 and IgG4 autoimmunity are similar, such as B cell tolerance defects resulting in autoreactive naïve B cells (234, 319–321) and T cell-assisted autoantigen affinity maturation, as evidenced by the presence of somatic mutations in most ASCs. In further support of the role of T cell help, autoreactive T cells have been observed in both IgG1 and IgG4 autoantibody-mediated disorders (264, 322, 323).

Differences between IgG4 and IgG1 autoimmune responses are not clinically trivial and can inform therapeutic decisions, especially since IgG4 autoantibody pathology responds impressively well to rituximab induction. More specifically, in IgG4 autoantibody-mediated disorders, prompt induction with rituximab 375 mg/m^2^ once a week for 4 to 6 weeks can result in a long-lasting favorable response and is highly recommended. The same induction can be applied in IgG1 disorders. It is important to obtain a pre-rituximab baseline and a post-B cell depletion autoantibody titer in all patients at regular intervals. In the case of a persistence of high titers of either IgG1 or, less frequently, IgG4 autoantibodies, which indicates the presence of LLPCs, repeated rituximab (or other CD20-depleting drug) dosing can be applied to enforce a deeper depletion of lymph node B cells and prevent the formation of new LLPCs, while existing ones slowly wane. An alternative strategy in such refractory cases is the administration of inebilizumab (or other CD19 depleting drug), which would neutralize CD19^+^ CD20^-^ ASCs that lie more towards the LLPC end of the ASC spectrum, or the application of daratumumab, an anti-CD38 agent more broadly targeting LLPCs. In the case that follicular or extrafollicular reactions within the CNS are suspected (e.g., based on advanced 7T MRI), it would be reasonable to apply an agent that, in contrast to monoclonal antibodies, can penetrate the blood-brain barrier and target B cells, such as a Bruton tyrosine kinase inhibitor. Finally, the application of new therapeutic avenues such as blockade of B-T cell interaction (CD40L) or IL-4 in polyrefractory cases warrants investigation. Ultimately and ideally, all these novel approaches should be tested in clinical trials prior to routine application.

Our review is not without limitations. First, many of the studies we reference were biased by the use of other immunosuppressants in addition to rituximab and did not have appropriate controls groups, since it is extremely hard to perform randomized controlled trials for rare disorders. Moreover, many of the diseases are aggressive and life-threatening and justify use of more than one immunosuppressant. Second, many of the studies presented and discussed relied on peripheral blood samples, which are easily accessible but not always representative of immunopathological procedures within secondary lymphoid organs or potential tertiary lymphoid structures within the CNS and PNS.

Overall, the differences between IgG1 and IgG4 autoimmune responses lead to many interesting new questions that could be explored in future investigations. Is the IgG4 autoimmune response a purely extrafollicular one? Can secondary or tertiary lymphoid structures be located in IgG4 autoantibody-mediated disorders? What are the different features of IgG1 and IgG4 response in the human lymph node? Does the memory cell compartment differ in IgG1 and IgG4 disorders? Is chronic antigenic stimulation necessary for emergence of autoimmunity of the IgG4 type and if yes, is it amenable to tolerization strategies? In IgG1 autoimmune responses, can LLPCs survive in the brain as they do in the bone marrow, and if yes, how can one target all LLPC niches therapeutically? Answering these questions would involve a detailed investigation of IgG1 and IgG4 differential maturation pathways in either extrafollicular spaces or in GC and would improve our understanding of disease mechanisms as well as facilitate development of new therapeutic avenues. New concepts could involve drugs that target crucial cellular interactions that are perceived to be responsible for B cell differentiation and maturation—in particular the T cell and B cell interaction, always keeping in mind that it is the aberrant and not the physiological response that needs to be stopped.

## Author Contributions

CZ: drafting and editing. AV: drafting and editing. PS: concept, design, drafting, and editing. All authors contributed to the article and approved the submitted version.

## Conflict of Interest

The authors declare that the research was conducted in the absence of any commercial or financial relationships that could be construed as a potential conflict of interest.
